# Supplementary material for: Hot spot prediction in protein-protein interactions by an ensemble system
Source: BMC Syst Biol. 2018 Dec 31;12(Suppl 9):132. doi: 10.1186/s12918-018-0665-8 (PMC6311905; doi:10.1186/s12918-018-0665-8)
Supplement: Supplementary file 2 — All relevant MATLAB code. A simple Matlab implement of our predictor. (ZIP 19,742 kb) [file 12918_2018_665_MOESM2_ESM.zip › Additional file 2/Read ASA/ASA website/dbMPIKT/1YY9D.html]

 NetSurfP - Protein Surface Accessibility and Secondary Structure Predictions

  

|  |  |  |
| --- | --- | --- |
|  |  | NetSurfP - Protein Surface Accessibility and Secondary Structure Predictions  Technical University of Denmark |

  


---

```
# For publication of results, please cite:
# A generic method for assignment of reliability scores applied to solvent accessibility predictions.
# Bent Petersen, Thomas Nordahl Petersen, Pernille Andersen, Morten Nielsen and Claus Lundegaard
# BMC Structural Biology 2009, 9:51 doi:10.1186/1472-6807-9-51
#
# Column 1: Class assignment - B for buried or E for Exposed - Threshold: 25% exposure, but not based on RSA
# Column 2: Amino acid
# Column 3: Sequence name
# Column 4: Amino acid number
# Column 5: Relative Surface Accessibility - RSA
# Column 6: Absolute Surface Accessibility
# Column 7: Z-fit score for RSA prediction
# Column 8: Probability for Alpha-Helix
# Column 9: Probability for Beta-strand
# Column 10: Probability for Coil
E Q  1YY9D                  1    0.801 142.987   1.268   0.003   0.003   0.994
B V  1YY9D                  2    0.212  32.569  -0.219   0.004   0.616   0.381
E Q  1YY9D                  3    0.435  77.637   1.237   0.002   0.816   0.182
B L  1YY9D                  4    0.152  27.776   0.727   0.002   0.816   0.182
E K  1YY9D                  5    0.426  87.731   0.620   0.021   0.756   0.223
B Q  1YY9D                  6    0.237  42.275   0.569   0.004   0.420   0.576
E S  1YY9D                  7    0.453  53.103  -0.644   0.020   0.205   0.775
E G  1YY9D                  8    0.497  39.114  -1.867   0.018   0.088   0.893
E P  1YY9D                  9    0.512  72.582  -2.235   0.018   0.047   0.935
E G  1YY9D                 10    0.516  40.586  -1.715   0.020   0.205   0.775
E L  1YY9D                 11    0.388  71.116  -0.794   0.022   0.359   0.619
E V  1YY9D                 12    0.282  43.267   0.641   0.021   0.451   0.528
E Q  1YY9D                 13    0.410  73.190   1.253   0.020   0.205   0.775
E P  1YY9D                 14    0.457  64.862  -0.469   0.005   0.045   0.951
E S  1YY9D                 15    0.613  71.820  -1.833   0.005   0.045   0.951
E Q  1YY9D                 16    0.461  82.299  -0.450   0.004   0.138   0.858
E S  1YY9D                 17    0.430  50.384  -0.462   0.004   0.420   0.576
B L  1YY9D                 18    0.151  27.557   0.933   0.002   0.816   0.182
E S  1YY9D                 19    0.420  49.247   0.802   0.001   0.900   0.099
B I  1YY9D                 20    0.078  14.411   0.681   0.001   0.900   0.099
B T  1YY9D                 21    0.184  25.535   0.801   0.001   0.900   0.099
B C  1YY9D                 22    0.040   5.644   0.050   0.001   0.959   0.040
B T  1YY9D                 23    0.288  39.973   0.660   0.001   0.959   0.040
B V  1YY9D                 24    0.093  14.279  -0.433   0.001   0.900   0.099
B S  1YY9D                 25    0.167  19.584  -0.631   0.004   0.616   0.381
B G  1YY9D                 26    0.234  18.408  -2.055   0.004   0.197   0.799
B F  1YY9D                 27    0.227  45.479  -0.922   0.005   0.336   0.660
B S  1YY9D                 28    0.163  19.150  -1.335   0.004   0.514   0.481
B L  1YY9D                 29    0.114  20.855  -0.767   0.021   0.451   0.528
B T  1YY9D                 30    0.198  27.518  -1.512   0.021   0.279   0.699
B N  1YY9D                 31    0.263  38.489  -1.460   0.018   0.088   0.893
B Y  1YY9D                 32    0.118  25.195  -0.307   0.019   0.141   0.840
B G  1YY9D                 33    0.095   7.461  -0.052   0.022   0.359   0.619
B V  1YY9D                 34    0.031   4.811   0.269   0.023   0.655   0.322
B H  1YY9D                 35    0.053   9.659  -0.098   0.021   0.756   0.223
B W  1YY9D                 36    0.039   9.283   0.183   0.021   0.756   0.223
B V  1YY9D                 37    0.041   6.317   0.244   0.018   0.846   0.136
B R  1YY9D                 38    0.118  26.976   0.337   0.021   0.756   0.223
B Q  1YY9D                 39    0.099  17.753   0.623   0.022   0.359   0.619
B S  1YY9D                 40    0.230  26.933   0.819   0.005   0.045   0.951
E P  1YY9D                 41    0.364  51.637   0.709   0.005   0.015   0.979
E G  1YY9D                 42    0.572  45.032  -1.722   0.018   0.019   0.964
E K  1YY9D                 43    0.424  87.176   0.632   0.018   0.019   0.964
E G  1YY9D                 44    0.432  33.991  -0.423   0.018   0.088   0.893
B L  1YY9D                 45    0.168  30.816   1.004   0.005   0.336   0.660
B E  1YY9D                 46    0.102  17.802  -0.051   0.003   0.718   0.279
B W  1YY9D                 47    0.051  12.362   0.444   0.001   0.900   0.099
B L  1YY9D                 48    0.025   4.596   0.851   0.001   0.959   0.040
B G  1YY9D                 49    0.019   1.511  -0.118   0.001   0.959   0.040
B V  1YY9D                 50    0.102  15.708  -0.099   0.001   0.959   0.040
B I  1YY9D                 51    0.033   6.087   0.099   0.001   0.959   0.040
B W  1YY9D                 52    0.183  43.891   0.684   0.004   0.616   0.381
E S  1YY9D                 53    0.391  45.860  -1.034   0.004   0.138   0.858
E G  1YY9D                 54    0.611  48.078  -2.403   0.005   0.015   0.979
E G  1YY9D                 55    0.453  35.675  -2.099   0.016   0.005   0.979
E N  1YY9D                 56    0.483  70.682  -0.673   0.018   0.047   0.935
E T  1YY9D                 57    0.363  50.320  -0.441   0.019   0.141   0.840
E D  1YY9D                 58    0.434  62.539  -0.062   0.020   0.205   0.775
B Y  1YY9D                 59    0.223  47.741   0.339   0.056   0.142   0.802
B N  1YY9D                 60    0.246  35.985  -0.503   0.052   0.084   0.864
E T  1YY9D                 61    0.624  86.479  -0.722   0.053   0.043   0.903
E P  1YY9D                 62    0.377  53.567  -0.059   0.018   0.088   0.893
B F  1YY9D                 63    0.086  17.340  -0.497   0.020   0.205   0.775
E T  1YY9D                 64    0.373  51.763   0.683   0.021   0.279   0.699
B S  1YY9D                 65    0.178  20.873  -1.835   0.004   0.420   0.576
B R  1YY9D                 66    0.197  45.067   0.554   0.002   0.816   0.182
B L  1YY9D                 67    0.051   9.356  -0.358   0.001   0.900   0.099
B S  1YY9D                 68    0.121  14.158   0.668   0.001   0.959   0.040
B I  1YY9D                 69    0.046   8.566   0.729   0.001   0.959   0.040
B N  1YY9D                 70    0.116  17.041   0.184   0.002   0.816   0.182
E K  1YY9D                 71    0.276  56.711   0.642   0.004   0.616   0.381
B D  1YY9D                 72    0.266  38.345  -0.722   0.004   0.420   0.576
E N  1YY9D                 73    0.451  65.997  -0.233   0.018   0.088   0.893
B S  1YY9D                 74    0.250  29.253  -1.940   0.018   0.088   0.893
E K  1YY9D                 75    0.524 107.787   0.882   0.019   0.141   0.840
E S  1YY9D                 76    0.264  30.906  -0.312   0.005   0.336   0.660
B Q  1YY9D                 77    0.208  37.077   1.181   0.002   0.816   0.182
B V  1YY9D                 78    0.067  10.236  -0.569   0.001   0.959   0.040
B F  1YY9D                 79    0.179  36.026   0.524   0.001   0.959   0.040
B F  1YY9D                 80    0.050  10.015   0.765   0.001   0.959   0.040
B K  1YY9D                 81    0.181  37.191  -0.191   0.001   0.900   0.099
B M  1YY9D                 82    0.083  16.648  -0.964   0.004   0.616   0.381
E N  1YY9D                 83    0.408  59.717  -0.031   0.005   0.336   0.660
E S  1YY9D                 84    0.403  47.290  -0.751   0.004   0.197   0.799
B L  1YY9D                 85    0.107  19.628   0.336   0.004   0.085   0.910
E Q  1YY9D                 86    0.492  87.925   1.124   0.005   0.045   0.951
E S  1YY9D                 87    0.422  49.412  -0.687   0.052   0.084   0.864
E N  1YY9D                 88    0.447  65.382  -0.625   0.053   0.043   0.903
B D  1YY9D                 89    0.182  26.197  -0.880   0.018   0.088   0.893
B T  1YY9D                 90    0.122  16.977   0.880   0.021   0.451   0.528
B A  1YY9D                 91    0.037   4.044  -0.082   0.018   0.846   0.136
B I  1YY9D                 92    0.066  12.192   1.018   0.011   0.918   0.071
B Y  1YY9D                 93    0.033   6.967   0.961   0.011   0.918   0.071
B Y  1YY9D                 94    0.051  10.856   0.934   0.018   0.846   0.136
B C  1YY9D                 95    0.016   2.260   1.531   0.021   0.756   0.223
B A  1YY9D                 96    0.028   3.075   0.187   0.023   0.655   0.322
B R  1YY9D                 97    0.135  30.823   0.026   0.022   0.552   0.426
B A  1YY9D                 98    0.254  28.002  -0.505   0.021   0.279   0.699
B L  1YY9D                 99    0.280  51.250  -0.558   0.019   0.141   0.840
E T  1YY9D                100    0.415  57.616  -1.914   0.018   0.088   0.893
E Y  1YY9D                101    0.312  66.589  -1.652   0.018   0.088   0.893
E Y  1YY9D                102    0.390  83.386  -1.472   0.018   0.088   0.893
E D  1YY9D                103    0.306  44.051  -1.822   0.018   0.088   0.893
B Y  1YY9D                104    0.213  45.561  -0.800   0.018   0.088   0.893
B E  1YY9D                105    0.116  20.318  -1.058   0.020   0.205   0.775
B F  1YY9D                106    0.051  10.256  -0.018   0.020   0.205   0.775
B A  1YY9D                107    0.060   6.601  -0.605   0.021   0.279   0.699
B Y  1YY9D                108    0.060  12.865  -0.398   0.021   0.279   0.699
B W  1YY9D                109    0.049  11.736  -0.772   0.020   0.205   0.775
B G  1YY9D                110    0.049   3.880  -1.247   0.004   0.138   0.858
B Q  1YY9D                111    0.169  30.255  -0.155   0.004   0.138   0.858
B G  1YY9D                112    0.099   7.791  -1.413   0.005   0.336   0.660
B T  1YY9D                113    0.062   8.655  -0.318   0.004   0.616   0.381
B L  1YY9D                114    0.178  32.573  -0.007   0.001   0.900   0.099
B V  1YY9D                115    0.030   4.611   0.333   0.001   0.900   0.099
B T  1YY9D                116    0.079  10.957   0.182   0.001   0.900   0.099
B V  1YY9D                117    0.053   8.100   0.542   0.002   0.816   0.182
B S  1YY9D                118    0.141  16.537  -0.411   0.004   0.514   0.481
E A  1YY9D                119    0.324  35.738  -1.198   0.004   0.197   0.799
E A  1YY9D                120    0.494  54.450  -2.064   0.005   0.045   0.951
E S  1YY9D                121    0.558  65.339  -0.855   0.018   0.047   0.935
E T  1YY9D                122    0.477  66.215  -1.960   0.005   0.015   0.979
E K  1YY9D                123    0.598 122.906  -1.581   0.005   0.015   0.979
E G  1YY9D                124    0.514  40.436  -1.680   0.005   0.015   0.979
B P  1YY9D                125    0.347  49.253  -1.550   0.018   0.019   0.964
E S  1YY9D                126    0.490  57.487  -1.201   0.018   0.088   0.893
B V  1YY9D                127    0.254  39.055  -1.688   0.020   0.205   0.775
B F  1YY9D                128    0.159  31.972  -0.382   0.021   0.279   0.699
E P  1YY9D                129    0.580  82.359  -0.736   0.021   0.279   0.699
B L  1YY9D                130    0.311  57.017  -1.374   0.019   0.141   0.840
E A  1YY9D                131    0.464  51.133  -1.403   0.004   0.085   0.910
E P  1YY9D                132    0.447  63.429  -2.214   0.005   0.045   0.951
E S  1YY9D                133    0.302  35.383  -2.206   0.005   0.045   0.951
E S  1YY9D                134    0.356  41.747  -2.480   0.005   0.045   0.951
E K  1YY9D                135    0.649 133.458  -2.300   0.005   0.015   0.979
E S  1YY9D                136    0.437  51.193  -2.219   0.003   0.003   0.994
E T  1YY9D                137    0.382  53.011  -1.621   0.003   0.003   0.994
E S  1YY9D                138    0.511  59.924  -1.772   0.003   0.003   0.994
E G  1YY9D                139    0.687  54.051  -2.302   0.003   0.003   0.994
E G  1YY9D                140    0.393  30.898  -1.992   0.005   0.045   0.951
E T  1YY9D                141    0.408  56.548   0.169   0.005   0.336   0.660
B A  1YY9D                142    0.047   5.212  -0.054   0.002   0.816   0.182
B A  1YY9D                143    0.213  23.506   0.324   0.001   0.900   0.099
B L  1YY9D                144    0.042   7.654  -0.195   0.001   0.900   0.099
B G  1YY9D                145    0.047   3.738  -0.162   0.001   0.900   0.099
B C  1YY9D                146    0.024   3.370   0.532   0.002   0.816   0.182
B L  1YY9D                147    0.149  27.264   0.458   0.002   0.816   0.182
B V  1YY9D                148    0.052   7.946  -0.400   0.003   0.718   0.279
E K  1YY9D                149    0.304  62.430  -0.167   0.004   0.514   0.481
E D  1YY9D                150    0.372  53.548  -2.297   0.005   0.262   0.733
B Y  1YY9D                151    0.218  46.629  -0.768   0.005   0.262   0.733
E F  1YY9D                152    0.351  70.385  -0.371   0.004   0.197   0.799
B P  1YY9D                153    0.221  31.346  -1.258   0.004   0.138   0.858
E E  1YY9D                154    0.606 105.886  -0.817   0.004   0.138   0.858
E P  1YY9D                155    0.463  65.657  -1.301   0.004   0.197   0.799
B V  1YY9D                156    0.202  31.047   0.400   0.004   0.616   0.381
E T  1YY9D                157    0.379  52.595  -0.332   0.001   0.900   0.099
B V  1YY9D                158    0.067  10.267   0.220   0.001   0.900   0.099
E S  1YY9D                159    0.305  35.758   0.454   0.001   0.900   0.099
B W  1YY9D                160    0.072  17.340  -0.446   0.011   0.918   0.071
E N  1YY9D                161    0.370  54.153   0.105   0.003   0.718   0.279
E S  1YY9D                162    0.510  59.725  -0.609   0.004   0.420   0.576
E G  1YY9D                163    0.483  37.988  -0.698   0.018   0.088   0.893
E A  1YY9D                164    0.568  62.616  -0.231   0.018   0.019   0.964
E L  1YY9D                165    0.556 101.804  -0.743   0.018   0.047   0.935
E T  1YY9D                166    0.608  84.260  -2.056   0.018   0.088   0.893
E S  1YY9D                167    0.555  65.034  -0.159   0.004   0.085   0.910
E G  1YY9D                168    0.454  35.753  -0.469   0.004   0.138   0.858
B V  1YY9D                169    0.197  30.248  -0.352   0.004   0.420   0.576
E H  1YY9D                170    0.355  64.520   0.305   0.002   0.816   0.182
B T  1YY9D                171    0.277  38.475  -0.442   0.001   0.900   0.099
B F  1YY9D                172    0.186  37.350   0.923   0.002   0.816   0.182
B P  1YY9D                173    0.177  25.102  -1.115   0.004   0.616   0.381
B A  1YY9D                174    0.234  25.820  -1.253   0.003   0.718   0.279
B V  1YY9D                175    0.204  31.370  -1.507   0.001   0.900   0.099
E L  1YY9D                176    0.307  56.230  -0.400   0.002   0.816   0.182
E Q  1YY9D                177    0.416  74.333  -0.361   0.004   0.514   0.481
E S  1YY9D                178    0.732  85.790  -1.672   0.004   0.085   0.910
E S  1YY9D                179    0.731  85.661  -1.376   0.005   0.015   0.979
E G  1YY9D                180    0.410  32.259  -1.470   0.004   0.138   0.858
E L  1YY9D                181    0.375  68.736   0.534   0.004   0.616   0.381
B Y  1YY9D                182    0.145  30.944  -0.031   0.001   0.900   0.099
B S  1YY9D                183    0.158  18.541  -0.780   0.001   0.900   0.099
B L  1YY9D                184    0.116  21.185  -0.623   0.001   0.959   0.040
B S  1YY9D                185    0.197  23.088  -0.183   0.001   0.959   0.040
B S  1YY9D                186    0.043   4.981  -1.015   0.001   0.959   0.040
B V  1YY9D                187    0.222  34.060   0.509   0.001   0.959   0.040
B V  1YY9D                188    0.052   8.069   0.106   0.001   0.959   0.040
E T  1YY9D                189    0.276  38.295   0.859   0.001   0.900   0.099
B V  1YY9D                190    0.075  11.604   0.830   0.002   0.816   0.182
E P  1YY9D                191    0.368  52.276   1.154   0.022   0.552   0.426
E S  1YY9D                192    0.391  45.837  -0.271   0.021   0.279   0.699
E S  1YY9D                193    0.600  70.332  -0.788   0.021   0.279   0.699
E S  1YY9D                194    0.622  72.863   0.352   0.021   0.279   0.699
B L  1YY9D                195    0.198  36.254  -0.781   0.004   0.138   0.858
E G  1YY9D                196    0.752  59.190   0.604   0.005   0.015   0.979
E T  1YY9D                197    0.742 102.915   0.940   0.005   0.015   0.979
E Q  1YY9D                198    0.614 109.696   0.901   0.004   0.197   0.799
E T  1YY9D                199    0.525  72.748   1.300   0.002   0.816   0.182
B Y  1YY9D                200    0.074  15.814   0.479   0.001   0.959   0.040
E I  1YY9D                201    0.360  66.674   1.900   0.001   0.959   0.040
B C  1YY9D                202    0.026   3.678   0.023   0.001   0.959   0.040
E N  1YY9D                203    0.399  58.472   1.151   0.001   0.900   0.099
B V  1YY9D                204    0.073  11.220  -0.411   0.002   0.816   0.182
E N  1YY9D                205    0.561  82.057   0.731   0.002   0.816   0.182
B H  1YY9D                206    0.187  33.979   0.206   0.004   0.514   0.481
E K  1YY9D                207    0.654 134.487   0.447   0.005   0.045   0.951
E P  1YY9D                208    0.756 107.276  -1.217   0.005   0.015   0.979
E S  1YY9D                209    0.542  63.522  -0.523   0.005   0.045   0.951
E N  1YY9D                210    0.615  90.007  -0.099   0.005   0.045   0.951
E T  1YY9D                211    0.473  65.619   0.676   0.004   0.197   0.799
E K  1YY9D                212    0.575 118.319   1.368   0.004   0.514   0.481
B V  1YY9D                213    0.121  18.567  -0.100   0.003   0.718   0.279
E D  1YY9D                214    0.671  96.691   0.543   0.002   0.816   0.182
E K  1YY9D                215    0.297  61.052   1.101   0.002   0.816   0.182
E R  1YY9D                216    0.582 133.301   0.912   0.003   0.718   0.279
E V  1YY9D                217    0.255  39.224   0.676   0.004   0.420   0.576
E E  1YY9D                218    0.505  88.293   0.228   0.004   0.197   0.799
E P  1YY9D                219    0.493  69.957  -1.041   0.004   0.138   0.858
E K  1YY9D                220    0.564 116.097  -0.523   0.018   0.019   0.964
E S  1YY9D                221    0.899 105.316  -0.927   0.000   0.000   0.999

Explain the output.  Go back.
```

---
